# The RNA-binding protein RBM39 scaffolds an m⁶A-dependent RNA decay complex that destabilizes Tat transcripts and restricts HIV-1 reactivation

**DOI:** 10.1371/journal.pbio.3003486

**Published:** 2025-11-11

**Authors:** Xiaohui Deng, Siyi Xie, Mo Zhou, Quyu Yuan, Shuangxin Wu, Peiming Huang, Minghua Chen, Jianteng Zeng, Pengle Guo, Jie Qin, Cancan Chen, Jiaye Liu, Bingfeng Liu, Xin He, Liqin Sun, Hui Zhang, Linghua Li, Ting Pan

**Affiliations:** 1 Shenzhen Key Laboratory for Systems Medicine in Inflammatory Diseases, School of Medicine, Shenzhen Campus of Sun Yat-Sen University, Sun Yat-Sen University, Shenzhen, Guangdong, China; 2 Guangdong Institute of Intelligence Science and Technology, Zhuhai, Guangdong, China; 3 Infectious Disease Center, Guangzhou Eighth People’s Hospital, Guangzhou Medical University, Guangzhou, China; 4 Institute of Human Virology, Department of Pathogen Biology and Biosecurity, Key Laboratory of Tropical Disease Control of Ministry Education, Guangdong Engineering Research Center for Antimicrobial Agent and Immunotechnology, Zhongshan School of Medicine, Sun Yat-sen University, Guangzhou, Guangdong, China; 5 Medical Research Center, The Eighth Affiliated Hospital of Sun Yat-sen University, Shenzhen, Guangdong, China; 6 Department of Gastrointestinal Surgery, Shenzhen Third People’s Hospital, The Second Hospital Affiliated to Southern University of Science and Technology, Shenzhen, Guangdong, China; 7 Department of Pathology, The First Affiliated Hospital, Sun Yat-sen University, Guangzhou, Guangdong, China; 8 School of Public Health, Shenzhen University Health Science Center, Shenzhen, Guangdong, China; 9 Department Infectious Diseases, National Clinical Research Center for Infectious Diseases, Shenzhen Third People’s Hospital, Shenzhen, Guangdong, China; Ulm University Medical Center, GERMANY

## Abstract

The persistence of latent HIV-1 reservoirs remains a critical barrier to functional curing AIDS, as current latency-reversing agents (LRAs) exhibit limited clinical efficacy. While RNA modifications like N⁶-methyladenosine (m⁶A) regulate viral replication, their role in maintaining HIV-1 latency is poorly defined. Here, we identify the RNA-binding protein RBM39 as a scaffold organizing an m⁶A-dependent silencing complex that enforces viral latency. Through proteomic and functional analyses, we demonstrate that RBM39 recruits the m⁶A reader YTHDC1 and the RNA helicase DDX5, forming a tripartite complex that accelerates Tat RNA decay and enforces viral quiescence. Genetic or pharmacological degradation of RBM39 (using the clinically explored molecular glue *indisulam*) potently reactivates latent HIV-1 in J-Lat cell models, primary CD4⁺ T cells from people living with HIV-1 (PLWH), and synergizes with established LRAs (Bryostatin-1, JQ-1, SAHA) to broadly activate proviral reservoirs. Our work reveals a previously unrecognized host pathway in which RBM39-organized RNA decay complexes silence HIV-1 through epitranscriptomic regulation of Tat. In addition to establishing RBM39 as a promising therapeutic target for addressing the limitations of current “shock and kill” strategies, our findings establish a novel mechanistic framework for m⁶A-dependent regulation of viral gene expression. This framework may serve as a valuable reference for investigating similar regulatory mechanisms in other latent viral infections or oncogenic processes where RNA methylation plays a pivotal role.

## Abstract 2

Latent HIV-1 reservoirs resist current reactivation therapies, hindering viral eradication. This study identified the RNA-binding protein RBM39 as a scaffold assembling an m⁶A-dependent RNA decay complex that degrades Tat transcripts enforcing and mantaining HIV-1 latency, establishing RBM39 as a promising therapeutic target.

## Introduction

Human Immunodeficiency Virus 1 (HIV-1), which causes Acquired Immune Deficiency Syndrome (AIDS), predominantly infects CD4^+^ T cells, leading to their progressive depletion and immune dysfunction. While active HIV-1 replication results in widespread CD4^+^ T cell destruction, the virus also establishes a latent reservoir in resting memory CD4^+^ T cells, enabling lifelong persistence and occasional reactivation to produce infectious virions [1,2]. This latent reservoir poses a significant barrier to achieving a functional cure for HIV-1. Current therapeutic strategies, such as the “shock and kill” approach, aim to reactivate latent HIV-1 using latency-reversing agents (LRAs), rendering infected cells visible to the immune system for elimination [3]. However, the limited clinical efficacy of existing LRAs underscores the urgent need to identify novel host factors that sustain viral latency and to develop more effective therapeutic targets.

Central to HIV-1 gene expression and latency is the viral *trans-*activator of transcription (Tat) protein. Tat binds to the *trans-*activation response element (TAR) in nascent viral RNA and recruits the positive transcription elongation factor b (P-TEFb) complex to the HIV-1 long terminal repeat (LTR) promoter, thereby enhancing transcriptional elongation and viral replication [4,5]. Beyond its canonical role, Tat interacts with host factors such as the E3 ligase TRAF6, activating the NF-κB pathway and modulating immune responses [6]. Recent advances have highlighted Tat’s potential as a target for latency reversal. For instance, lipid nanoparticles delivering Tat mRNA (Tat-LNP), in combination with histone deacetylase inhibitors, have demonstrated superior reactivation of latent HIV-1 compared to conventional treatments [7]. These findings highlight Tat’s potential as a therapeutic target for latency reversal, yet the mechanisms regulating its stability and activity during latency remain poorly understood. Moreover, recent studies implicate RNA-binding proteins (RBPs) and epitranscriptomic marks such as m⁶A in modulating viral replication [8], yet their interplay with Tat stability during latency is unexplored.

RNA-binding Motif Protein 39 (RBM39), a splicing factor implicated in cancer progression, interacts with transcriptional regulators such as AP-1 and NF-κB, suggesting a potential role in HIV-1 latency [9–13]. Similarly, m⁶A modifications, mediated by reader proteins like YTHDC1, influence viral replication cycles and host immune responses [14,15]. YTHDC1, a nuclear m⁶A reader, regulates RNA metabolism by binding m⁶A-modified transcripts, yet its role in HIV-1 latency remains unexplored [16–18].

Here, we address these critical gaps by elucidating a novel mechanism by which RBM39 enforces HIV-1 latency through m⁶A-dependent Tat degradation. We demonstrate that RBM39 enhances m⁶A modifications on Tat RNA, recruiting YTHDC1 and the RNA helicase DDX5 to form a tripartite complex that accelerates Tat RNA decay. Importantly, we show that pharmacological degradation of RBM39 using the molecular glue *indisulam* reactivates latent HIV-1 in cell models and primary CD4⁺ T cells from people living with HIV-1 (PLWH). These findings not only reveal a previously unrecognized epitranscriptomic pathway controlling Tat stability but also establish RBM39 as a therapeutic target for enhancing the “shock and kill” strategy.

## Results

### RBM39 is identified as a key host factor regulating Tat stability

**Fig 1:**
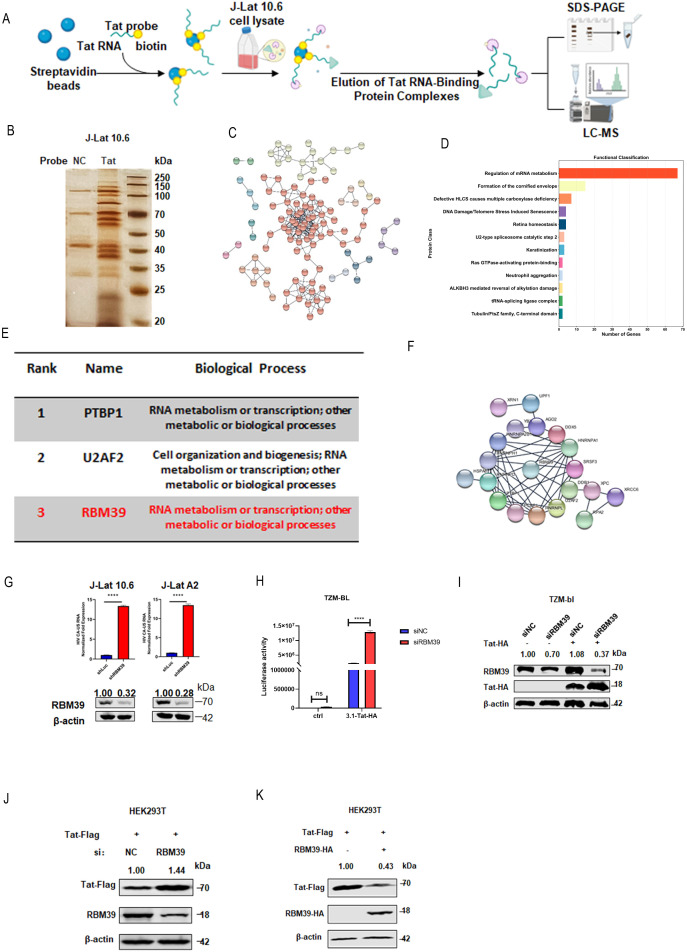
RBM39 is identified as a key host factor regulating Tat stability. **(A)** Schematic diagram of the experimental strategy for identifying HIV-1 Tat RNA-binding proteins in J-Lat 10.6 cells. **(B)** Silver staining of proteins pulled down using Tat RNA as bait. **(C)** Protein–protein interaction network constructed from Tat-associated proteomic data (*n* = 181) using the STRING database. The network was subjected to K-means clustering (*k* = 12), with each color representing a distinct protein cluster. **(D)** KEGG pathway enrichment analysis of the 12 protein clusters was performed using the STRING database, revealing the top functional categories. The horizontal bar chart displays the number of genes associated with each pathway, with “Regulation of mRNA metabolism” being the most enriched category (>60 genes). **(E)** Table showing the top three proteins ranked by bottleneck analysis, along with their associated biological processes. **(F)** Interaction network of the top 20 proteins ranked by bottleneck centrality using the CytoHubba plugin, highlighting RBM39 and its direct interacting partners within the network. **(G)** J-Lat 10.6 and J-Lat A2 cells were infected with shLuc or shRBM39 lentiviruses, respectively. After 48 hours of infection, the relative levels of HIV CA-US RNA were quantified by RT–qPCR (upper panel), and endogenous RBM39 expression was assessed by western blotting (lower panel). Data are presented as means ± SEM from triplicate measurements. *P*-values were calculated using an unpaired Student *t* test. ****P < 0.0001. **(H–I)** TZM-bl cells were transfected with siNC or siRBM39. Twenty-four hours after siRNA transfection, the cells were subsequently transfected with the Tat-HA plasmid. At 48 hours post-transfection, cells were harvested for luciferase activity assays (H) and western blot analysis (I). Data are presented as means ± SD from triplicate experiments. *P*-values were calculated using a two-way ANOVA with Sidak’s multiple comparisons test. ****P < 0.0001; ns, not significant. **(J)** HEK293T cells were transfected with siNC or siRBM39. Twenty-four hours after siRNA transfection, the cells were subsequently transfected with the Tat-Flag plasmid. At 24 hours post-transfection, cells were lysed and subjected to western blot analysis using the indicated antibodies. **(K)** HEK293T cells were co-transfected with Tat-Flag and RBM39-HA plasmids. At 48 hours post-transfection, cells were lysed and subjected to western blot analysis using the indicated antibodies. The underlying data for this figure can be found in the ‘Raw Data’ file in https://doi.org/10.5281/zenodo.17470926.

Given the pivotal role of Tat in HIV-1 latency, we sought to identify proteins that interact with Tat and contribute to the maintenance of viral quiescence. We performed RNA pull-down assays in latently HIV-1-infected J-Lat 10.6 cells using biotinylated RNA probes specific to the Tat RNA and a control RNA probe (Fig 1A). Cell lysates were incubated with streptavidin agarose resin pre-bound to these probes, and the enriched Tat RBPs were separated by SDS-PAGE and analyzed by liquid chromatography–mass spectrometry (LC–MS/MS) (Fig 1B).

To elucidate the functional relationships among these Tat-associated proteins, we performed a comprehensive protein-protein interaction network analysis using the STRING database. We constructed an interaction network from the 181 proteins identified in J-Lat 10.6 cells and applied K-means clustering with *k* = 12 to group functionally related protein modules, represented by distinct colors (Fig 1C). KEGG pathway enrichment analysis of this network revealed significant enrichment in several key biological processes (Fig 1D). The most prominently enriched pathway was “Regulation of mRNA metabolism,” involving over 60 genes, which aligns with the known role of Tat in transcriptional regulation.

Among the top three proteins identified in our analysis (Fig 1E), the top-ranked PTBP1 (polypyrimidine tract binding protein (1) and the second-ranked U2AF2 (U2 small nuclear RNA auxiliary factor (2) have well-established roles in HIV-1 biology. Specifically, PTBP1 upregulation, caused by gp120, leads to the splicing of pyruvate kinase M (PKM) into its isoforms PKM1 and PKM2 [19]. U2AF2 (also called U2AF65) is antagonized by the HIV-1 hnRNP A/B-dependent exonic splicing silencer ESSV, which interferes with its binding to viral polypyrimidine tracts [20]. Given these prior findings, we focused on the third-ranked hub protein, RBM39, which has been broadly implicated in post-transcriptional regulation and interact with a wide range of RNA processing factors [21]. To identify key hub proteins within the network, we employed bottleneck centrality analysis using the CytoHubba plugin and focused on the top 20 ranked proteins (Fig 1F). This analysis confirmed RBM39 as a central node with extensive interactions, connecting to multiple proteins including HNRNPA1, SRSF3, DDX5, and other RBPs, highlighting its potential importance in the Tat-mediated regulatory network.

To explore the functional role of RBM39 in HIV-1 latency, we observed that depletion of RBM39 via shRNA robustly reactivated latent pseudotyped HIV-1 in J-Lat 10.6 and J-Lat A2 cells (Fig 1G), establishing RBM39 as a key enforcer of viral quiescence. To further validate these findings and investigate the underlying mechanism, we conducted transfection experiments on TZM-bl cells, which serve as a reporter cell line for Tat activity. We co-transfected these cells with Tat-HA and either a control siRNA (siNC) or siRBM39. We found that knockdown of endogenous RBM39 significantly enhanced HIV-1 LTR-driven luciferase activity in the presence of Tat (Fig 1H). Western blotting confirmed that this effect was accompanied by a notable increase in Tat protein levels (Fig 1I). To corroborate these findings, we performed additional experiments in HEK293T cells. We overexpressed Tat-Flag while either knockdown endogenous RBM39 or overexpressing RBM39-HA. Consistent with the results in TZM-bl cells, Tat expression was upregulated upon RBM39 knockdown and downregulated upon RBM39 overexpression (Fig 1J and 1K). Collectively, these findings demonstrate that RBM39 is a key regulator of Tat protein stability and expression, and that it maintains HIV-1 latency by controlling Tat protein levels.

### RBM39 mediates Tat mRNA degradation via m⁶A methylation

**Fig 2:**
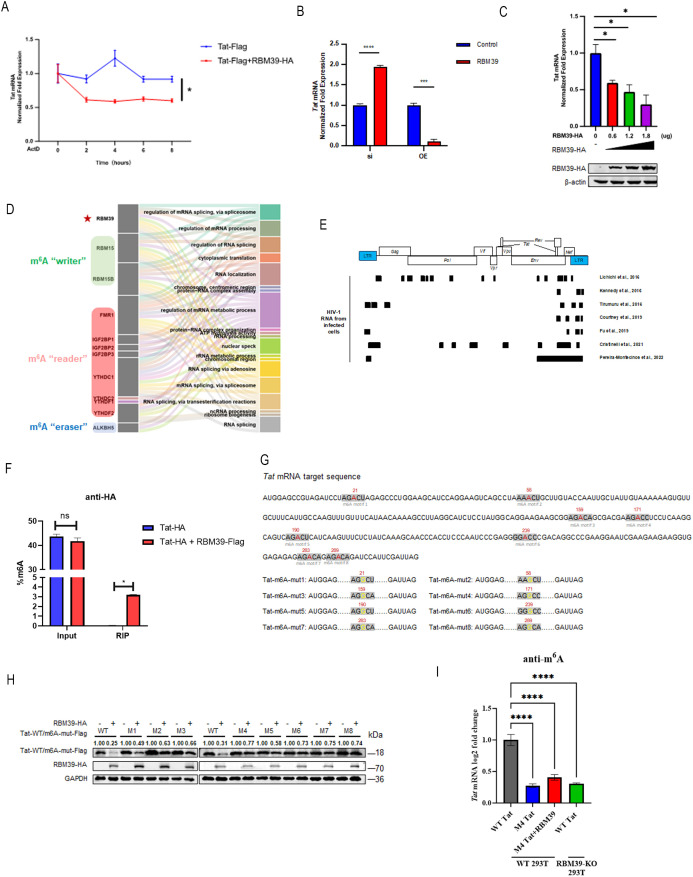
RBM39 mediates m⁶A methylation to regulate Tat mRNA stability. **(A)** HEK293T cells were transfected with Tat-Flag and RBM39-HA. At 24 hours post-transfection, Tat mRNA stability was assessed by RT-qPCR following treatment with the transcriptional inhibitor Actinomycin D. The relative mRNA level at 0 hours after ActD treatment was normalized to 1. Data are presented as means ± SEM from triplicate measurements. *P*-values were calculated using a paired *t* test. *P < 0.05. **(B)** HEK293T cells were transfected with siRBM39 or RBM39-HA. Twenty-four hours after siRNA transfection, the cells were subsequently transfected with Tat-HA plasmids. At 24 hours post-transfection, cells were lysed for RNA extraction, and the relative Tat RNA levels were quantified using RT-qPCR. Data are presented as means ± SEM from triplicate measurements. *P*-values were calculated using an unpaired Student *t* test. ***P < 0.001; ****P < 0.0001. **(C)** HEK293T cells were transfected with Tat-Flag and increasing amounts of RBM39-HA (0.6, 1.2, and 1.8 µg). At 24 hours post-transfection, cells were lysed for RNA extraction, and the relative Tat RNA levels were quantified using RT-qPCR (upper panel). Western blot analysis was performed to assess RBM39-HA expression using the indicated antibodies (lower panel). The relative mRNA level without RBM39-HA transfection was normalized to 1. Data are presented as means ± SEM from triplicate measurements. *P*-values were calculated using an unpaired Student *t* test. *P < 0.05. **(D)** The Sankey diagram illus*t*rates the associations between 12 m⁶A-related proteins and their enriched biological pathways. **(E)** A proportional schematic diagram illustrates the locations of m⁶A peaks along the HIV-1 genome. The genome sequence of the HIV-1 NL4-3 strain was used as a reference for aligning findings from various studies. Black squares indicate the predicted positions of m⁶A peaks, inferred from diverse sequencing techniques applied to RNA extracted from HIV-1-infected cells. **(F)** The EpiQuik m⁶A RNA Methylation Quantification Kit was used to measure m⁶A levels in total RNA and in m⁶A-modified RNA enriched by RIP from HEK293T cells transfected with Tat-Flag and Vector-Flag or Tat-Flag and RBM39-HA plasmids. Data are presented as means ± SD from triplicate measurements. *P*-values were calculated using a two-way ANOVA with Sidak’s multiple comparisons test. *P < 0.05; ns, not significant. **(G)** A schematic diagram of the Tat mRNA sequence containing 8 DRACH motifs (highlighted in gray) is shown. The adenine (A) marked in red is the direct target of m⁶A modification, undergoing methylation at the N⁶ position. To block m⁶A methylation, this adenine was mutated to guanine (G). The diagram illustrates individual mutations at all eight m⁶A methylation sites. Both the wild-type Tat sequence (WT m⁶A motif) and the mutated Tat sequence (mut m⁶A motif) were cloned into the pCDNA3.1 expression vector with a Flag tag. **(H)** HEK293T cells were transfected with Flag-tagged wild-type Tat or various Tat mutants, together with HA-tagged RBM39. At 24 hours post-transfection, cells were lysed and subjected to western blot analysis using the indicated antibodies. **(I)** Assessment of Tat m⁶A modification levels was performed through RIP of m⁶A-modified RNA isolated from HEK293T cells transfected with WT Tat-Flag (gray), M4 Tat-Flag (blue), or M4 Tat-Flag and RBM39-HA (red), as well as RBM39-KO HEK293T cells transfected with WT Tat-Flag (green) plasmids, followed by reverse transcription-polymerase chain reaction (RT-PCR) analysis. Data are presented as means ± SD from triplicate measurements. *P*-values were calculated using a one-way ANOVA with Dunnett’s multiple comparisons test. ****P < 0.0001; ns, not significant. The underlying data for this figure can be found in the ‘Raw Data’ file in https://doi.org/10.5281/zenodo.17470926.

Given the involvement of RBM39 in mRNA metabolism, we next investigated whether its regulation of Tat stability occurs at the RNA level, specifically by influencing Tat mRNA stability. To assess the effect of RBM39 on Tat mRNA decay, we treated cells with the transcriptional inhibitor actinomycin D (ActD) and performed reverse transcription quantitative polymerase chain reaction (RT-qPCR). These experiments revealed that overexpression of RBM39 significantly accelerated the decay of Tat mRNA (Fig 2A), suggesting a post-transcriptional regulatory mechanism. Furthermore, knocking down endogenous RBM39 with siRNA led to an increase in Tat mRNA level, while RBM39 overexpression resulted in a marked reduction (Fig 2B). Furthermore, we observed that this effect is dose-dependent, with Tat RNA expression being progressively downregulated in a dose-dependent manner upon overexpression of RBM39 (Fig 2C).

To further understand the molecular mechanism, we sought to identify proteins that interact with RBM39. We transfected HEK293T cells with Flag-tagged RBM39, enriched RBM39 and its interacting partners using anti-Flag beads, and analyzed the enriched proteins by LC–MS/MS. Our analysis revealed significant enrichment of m⁶A regulatory proteins bound to RBM39 (Fig 2D). Considering the previously reported abundance of m⁶A modification sites on the HIV-1 RNA genome (Fig 2E) and the overlap of the ninth m⁶A methylation peak of HIV-1 genomic RNA with the N-terminal sequence of Tat [22–27], we hypothesized that RBM39 might regulate Tat through m⁶A modification.

To test this hypothesis, we quantified m⁶A levels in HEK293T cells transfected with Tat-HA alone or co-transfected with RBM39-Flag. Remarkably, we observed a significant increase in m⁶A levels on RNA immunoprecipitated with HA-tagged Tat protein when RBM39 was co-expressed (Fig 2F). To pinpoint the specific m⁶A sites involved, we analyzed the Tat coding sequence and identified eight potential m⁶A modification motifs (DRACH, D = A/G/U; R = G/A; H = A/C/U), with AGACA being the most canonical site. We then constructed Tat mutants with disrupted m⁶A modification sites and co-expressed them with RBM39 (Fig 2G). Intriguingly, RBM39-mediated degradation of Tat was partially inhibited in the mutants compared to wild-type (wt) Tat (Fig 2H), indicating that m⁶A modification plays a critical role in RBM39-dependent Tat regulation. To confirm that RBM39 promotes methylation in a site-specific manner, we conducted m⁶A RIP experiments using our m⁶A-mutant Tat constructs (Fig 2I). Additionally, m⁶A RIP-qPCR assays were performed in RBM39-knockout (KO) cells, which revealed a significant reduction in the overall m⁶A levels on Tat RNA (Fig 2I). These findings indicate that RBM39 actively facilitates Tat methylation, rather than merely associating with pre-existing m⁶A modifications. The results show that the m⁶A signal on mutant Tat RNA remains substantially lower than that on wt Tat RNA, even under conditions of RBM39 overexpression. This confirms that RBM39-mediated promotion of methylation is dependent on the specific DRACH motifs.

### RBM39 recruits YTHDC1 to degrade Tat RNA via m⁶A modifications

**Fig 3:**
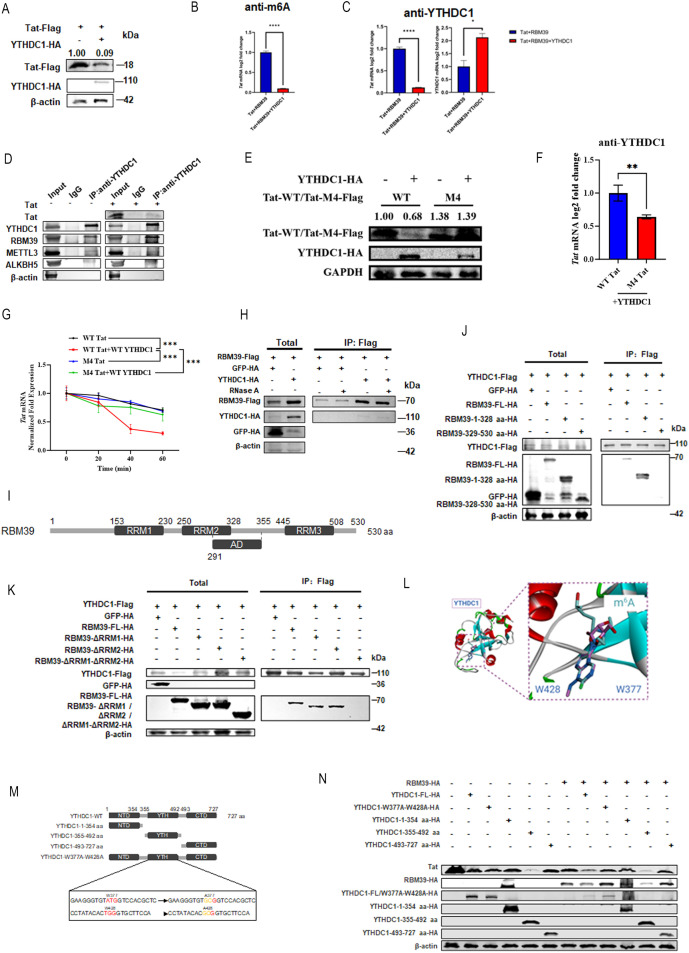
RBM39 recruits YTHDC1 to promote Tat degradation. **(A)** HEK293T cells were transfected with Tat-Flag alone or in combination with YTHDC1-HA plasmids. At 24 hours post-transfection, cells were lysed and subjected to western blot analysis using the indicated antibodies. **(B)** Assessment of Tat m⁶A modification levels through RIP of m⁶A-modified RNA isolated from HEK293T cells transfected with Tat-Flag, RBM39-HA, and YTHDC1-HA plasmids, followed by qRT-PCR analysis. Data are presented as means ± SEM from triplicate measurements. *P*-values were calculated using an unpaired Student *t* test. ****P < 0.0001. **(C)** Detection of Tat and YTHDC1 binding by immunoprecipitation of YTHDC1-associated RNA from HEK293T cells transfected with Tat-Flag, RBM39-HA, and YTHDC1-HA plasmids, followed by qRT-PCR analysis. Data are presented as means ± SEM from triplicate measurements. *P*-values were calculated using an unpaired Student *t* test. *P < 0.05; ****P < 0.0001. **(D)** HEK293T cells were transfected with Tat-Flag and Vector-Flag plasmids. At 48 hours post-transfection, cell lysates were immunoprecipitated with IgG (control) or anti-YTHDC1 antibody and analyzed by immunoblotting with the indicated antibodies. **(E)** HEK293T cells were transfected with Flag-tagged wild-type Tat or the Tat mutant M4, together with HA-tagged YTHDC1. At 24 hours post-transfection, cells were lysed and subjected to western blot analysis using the indicated antibodies. **(F)** Detection of WT Tat or M4 Tat and YTHDC1 binding was performed by immunoprecipitation of YTHDC1-associated RNA from HEK293T cells transfected with WT Tat-Flag or M4 Tat-Flag and YTHDC1-HA plasmids, followed by qRT-PCR. Data are presented as means ± SD from triplicate measurements. *P*-values were calculated using an unpaired Student *t* test. **P < 0.01. **(G)** HEK293T cells were *t*ransfected with WT Tat-Flag (black), WT Tat-Flag and YTHDC1-HA (red), M4 Tat-Flag (blue), or M4 Tat-Flag and YTHDC1-HA (green). At 24 hours post-transfection, Tat mRNA stability was assessed by RT-qPCR following treatment with the transcriptional inhibitor Actinomycin D. Data are presented as means ± SD from triplicate measurements. *P*-values were calculated using a one-way ANOVA with Dunnett’s multiple comparisons test. ***P < 0.001; ns, not significant. **(H)** HEK293T cells were transfected with RBM39-Flag together with GFP-HA (control) or YTHDC1-HA plasmids. At 48 hours post-transfection, cell lysates were immunoprecipitated using Flag beads, treated with RNase A, and analyzed by immunoblotting with the indicated antibodies. **(I)** Schematic representation of RBM39. RRM, RNA recognition motif; AD, active domain. Numbers indicate amino acid positions. **(J, K)** Immunoprecipitation and immunoblot analysis of HEK293T cells transfected to express HA-tagged wild-type RBM39 or various RBM39 substitution mutants, together with Flag-tagged YTHDC1. **(L)** Superposition of the crystal structures of YTHDC1 (PDB ID: 4R3I). The protein structures are displayed in cartoon mode, and the conserved tryptophan (W) residues are shown as sticks. The conserved aromatic cage is highlighted with a purple dashed box. **(M)** Schematic representation of YTHDC1. NTD, N-terminal domain; YTH, YT521-B homology domain; CTD, C-terminal domain. Numbers indicate amino acid positions. **(N)** HEK293T cells were transfected with Tat-Flag and wild-type YTHDC1 or YTHDC1 mutants, together with Vector-HA or RBM39-HA. At 24 hours post-transfection, cells were lysed and subjected to western blot analysis using the indicated antibodies. The underlying data for this figure can be found in the ‘Raw Data’ file in https://doi.org/10.5281/zenodo.17470926.

N^6^-methyladenosine (m⁶A) is the most abundant internal modification in mRNA and is crucial for regulating mRNA metabolism and gene expression. This modification influences cellular processes by altering mRNA structure and affecting the binding of RBPs [28]. To further elucidate the molecular mechanism by which m⁶A regulate Tat expression, we systematically analyzed the effects of seven m^6^A-related proteins (writers, erasers, and readers) on Tat expression. Our findings revealed that YTHDC1, an m⁶A reader protein, was the most significant suppressor of Tat expression (S1 Fig). As YTHDC1 is the only reported reader protein known to function within the cell nucleus, where Tat also localizes and functions, we focused on its potential mechanism. First, we assessed the effect of YTHDC1 on Tat RNA. Overexpression of YTHDC1 recapitulated RBM39’s ability to suppress Tat expression (Fig 3A), suggesting that the recognition of m⁶A marks on Tat RNA promotes its degradation. RIP-qPCR analysis using either an m⁶A-specific antibody or a YTHDC1-specific antibody showed that YTHDC1 overexpression decreased the overall m⁶A modification levels on Tat RNA (Fig 3B and 3C). This seemingly counterintuitive result may suggest that the binding and degradation by YTHDC1 are so rapid that they reduce the detectable pool of m⁶A-modified Tat RNA.

To understand the protein-protein interactions within this regulatory axis, we performed immunoprecipitation experiments using an endogenous YTHDC1 antibody. We demonstrated that Tat significantly enhances the interaction between YTHDC1 and RBM39 (Fig 3D). In contrast, the interactions with METTL3 (a key component of the m⁶A “writer” complex) and ALKBH5 (a key component of the m⁶A “eraser” complex) were relatively weaker (Fig 3D). This aligns with prior reports that RBPs like RBM15/RBM15B direct site-specific m⁶A deposition via the WTAP-METTL3 complex [29]. Given that RBM39 belongs to the same RBM family, we hypothesized that it acts as a cofactor, physically bridging METTL3-mediated m⁶A modification with YTHDC1-dependent RNA decay. While METTL3’s catalytic activity is necessary for initiating these processes, its weaker physical association with YTHDC1 suggests a transient rather than a stable structural role within the final degradation machinery.

To further confirm the critical role of m⁶A sites in YTHDC1-mediated degradation, we employed an m⁶A site-mutant Tat construct (M4), as previously identified in Fig 2G. Co-expression of this mutant with YTHDC1 revealed a marked reduction in YTHDC1’s ability to degrade the Tat protein (Fig 3E). Under the same conditions, RIP assays showed significantly diminished binding of YTHDC1 to the mutated Tat RNA (Fig 3F). Furthermore, ActD chase experiments indicated that the m⁶A mutant Tat RNA exhibited greater resistance to YTHDC1-mediated degradation compared to the wt transcript (Fig 3G). Together, these findings consistently demonstrate that the regulatory effect of YTHDC1 on Tat RNA is significantly dependent on m⁶A sites.

Next, we systematically investigated the interaction mechanism between RBM39 and YTHDC1. Specifically, RNase-treated immunoprecipitation experiments confirmed that their interaction is RNA-independent (Fig 3H). We then generated a series of RBM39 truncation and deletion mutants based on its key domains (RRM1, RRM2, RRM3, and AD domain) (Fig 3I). Systematic domain mapping revealed that the RRM1 and RRM2 motifs of RBM39 are essential for its interaction with YTHDC1 (Fig 3J and 3K). To confirm that YTHDC1’s m⁶A-binding function is required for this process, we leveraged its known binding mechanism. The YTH domain of YTHDC1 (amino acid residues 355–492) contains two conserved tryptophan residues (W377 and W428), which form an aromatic cage for recognizing m⁶A-modified bases (Fig 3L) [30]. By functionally truncating YTHDC1 based on its N-terminal domain (NTD), YTH domain, and C-terminal domain (CTD), we constructed an m⁶A-binding-deficient mutant (YTHDC1-W377A/W428A) via alanine substitution of W377 and W428 (Fig 3M). Notably, while wt YTHDC1 promoted Tat degradation, the aromatic cage mutant (YTHDC1-W377A/W428A) markedly attenuated the RBM39-enhanced degradation effect (Fig 3N). Collectively, these findings demonstrate that the m⁶A reader function of YTHDC1 is indispensable for RBM39-mediated Tat degradation.

### RBM39 and YTHDC1 regulate Tat degradation via DDX5

**Fig 4:**
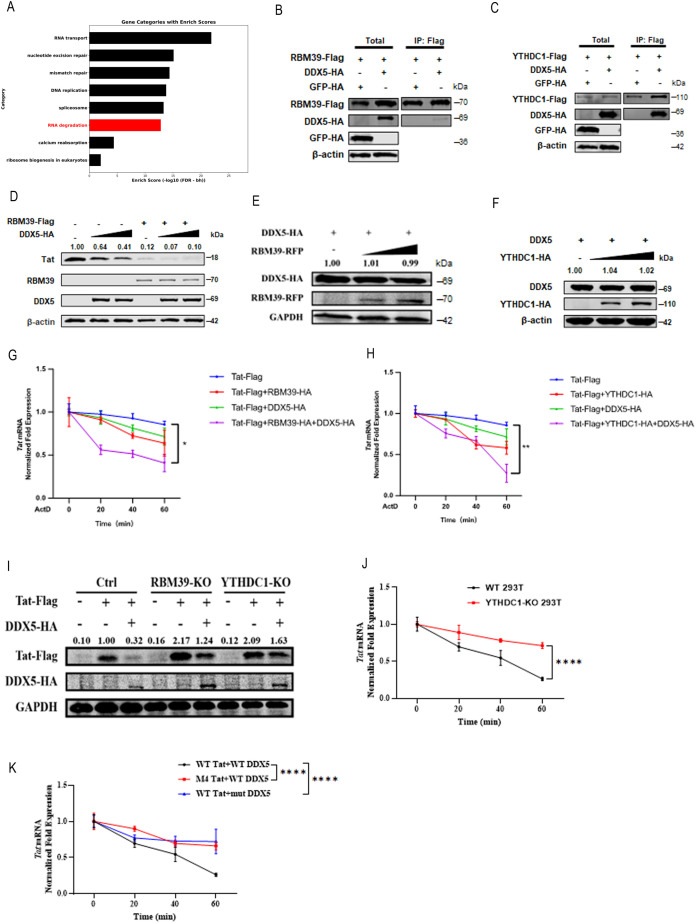
RBM39 and YTHDC1 mediate Tat degradation via DDX5. **(A)** HEK293T cells were transfected with Tat-HA and Vector-Flag or Tat-HA and RBM39-Flag plasmids. At 48 hours post-transfection, proteins were enriched using anti-Flag beads, separated by SDS-PAGE, and subjected to liquid chromatography-–mass spectrometry (LC–MS/MS) analysis. KEGG pathway analysis was performed on the enriched proteins. Members of the DDX family associated with RNA degradation were identified. **(B, C)** HEK293T cells were transfected with RBM39-Flag and GFP-HA or RBM39-Flag and DDX5-HA plasmids (B), or YTHDC1-Flag and GFP-HA or YTHDC1-Flag and DDX5-HA plasmids (C). At 48 hours post-transfection, cell lysates were immunoprecipitated using Flag beads and analyzed by immunoblotting with the indicated antibodies. **(D)** HEK293T cells were co-transfected with Tat-Flag and increasing amounts of DDX5-HA, together with Vector-HA or RBM39-HA. At 24 hours post-transfection, cells were lysed and subjected to western blot analysis using the indicated antibodies. **(E)** HEK293T cells were transfected with DDX5-HA and increasing amounts of RBM39-RFP plasmids. At 24 hours post-transfection, cells were lysed and western blot analysis was performed using the indicated antibodies. **(F)** HEK293T cells were transfected with DDX5-Flag and increasing amounts of YTHDC1-HA plasmids. At 24 hours post-transfection, cells were lysed and western blot analysis was conducted using the indicated antibodies. **(G, H)** HEK293T cells were transfected with Tat-Flag, RBM39-HA, and DDX5-HA (G), or Tat-Flag, YTHDC1-HA, and DDX5-HA (H). At 24 hours post-transfection, Tat mRNA stability was assessed by RT-qPCR following treatment with the transcriptional inhibitor Actinomycin D. Data are presented as means ± SEM from triplicate measurements. *P*-values were calculated using an unpaired Student *t* test. *P < 0.05; **P < 0.01. **(I)** Tat-Flag and DDX5-HA plasmids were co-transfected into wild-type HEK293T cells, RBM39-KO HEK293T cells, or YTHDC1-KO HEK293T cells. At 24 hours post-transfection, cells were lysed and western blot analysis was performed using the indicated antibodies. (J) Wild-type HEK293T cells were transfected with WT Tat-Flag, WT DDX5-HA, and YTHDC1-HA (black), or YTHDC1-KO HEK293T cells were transfected with WT Tat-Flag and WT DDX5-HA (red). At 24 hours post-transfection, Tat mRNA stability was assessed by RT-qPCR following treatment with the transcriptional inhibitor Actinomycin D. Data are presented as means ± SD from triplicate measurements. *P*-values were calculated using an unpaired Student *t* test. ****P < 0.0001. **(K)** HEK293T cells were transfected with WT Tat-Flag and WT DDX5-HA (black), M4 Tat-Flag and WT DDX5-HA (red), or WT Tat-Flag and mutant DDX5-HA (blue), together with YTHDC1-HA. At 24 hours post-transfection, Tat mRNA stability was assessed by RT-qPCR following treatment with Actinomycin D. Data are presented as means ± SD from triplicate measurements. *P*-values were calculated using an unpaired one-way ANOVA. ****P < 0.0001. The underlying data for this figure can be found in the ‘Raw Data’ file in https://doi.org/10.5281/zenodo.17470926.

To gain deeper insight into the mechanism by which RBM39 regulates Tat RNA decay, we performed a KEGG pathway analysis of the mass spectrometry data from Fig 2D. This analysis of RBM39-interacting proteins revealed several factors associated with RNA degradation, particularly members of the DEAD/H-box (DDX/DHX) RNA helicases family. Among these, DDX5, DDX21, and DHX36 were identified as high-confidence RBM39 partners in regulating Tat expression (Fig 4A). We focused on DDX5 because it has been previously implicated in RNA processing [31] and is known to form a complex with the methyltransferase METTL3, participating in RNA m⁶A modification, and to interact with the m⁶A reader YTHDC1 to promote the production of circRNAs [32–34]. Therefore, we further began by confirming the physical interactions. Co-IP experiments verified direct interactions between RBM39 and DDX5, as well as between YTHDC1 and DDX5 (Fig 4B and 4C). Subsequent functional assays revealed that DDX5 significantly potentiated RBM39-mediated Tat degradation in a dose-dependent manner (Fig 4D). Importantly, DDX5 expression levels were not influenced by either RBM39 or YTHDC1 (Fig 4E and 4F), indicating that DDX5 acts as a critical cofactor within the Tat degradation pathway rather than being a downstream target.

To further investigate the role of DDX5 in Tat degradation, we performed RT-qPCR analysis following treatment with the transcriptional inhibitor ActD. The results showed that co-overexpression of DDX5 with either RBM39 or YTHDC1 significantly accelerated the degradation of Tat mRNA (Fig 4G and 4H). To confirm DDX5’s dependency on these proteins, we used RBM39- or YTHDC1-KO HEK293T cells. Notably, in these KO backgrounds, DDX5 failed to promote Tat degradation (Fig 4I and 4J), confirming that both RBM39 and YTHDC1 are essential for DDX5’s function. This supports a model where DDX5, YTHDC1, and m⁶A-modified Tat RNA form a ternary complex to facilitate RNA decay. Moreover, using m⁶A site-mutant Tat construct (Tat-M4), we found that DDX5 was no longer able to effectively promote Tat RNA degradation (Fig 4K), indicating a clear dependence on m⁶A modification. Additionally, ActD chase assays with a helicase-deficient DDX5 mutant (DEAD box mutant) demonstrated that the loss of helicase activity completely abrogated DDX5’s ability to enhance Tat RNA decay (Fig 4K). These results collectively suggest that DDX5, RBM39, and YTHDC1 likely form a cooperative trimeric complex, where DDX5’s helicase activity, guided by RBM39 and YTHDC1, is crucial for the degradation of Tat RNA.

### RBM39 sustains HIV-1 latency and is a pharmacologically targetable hub

**Fig 5:**
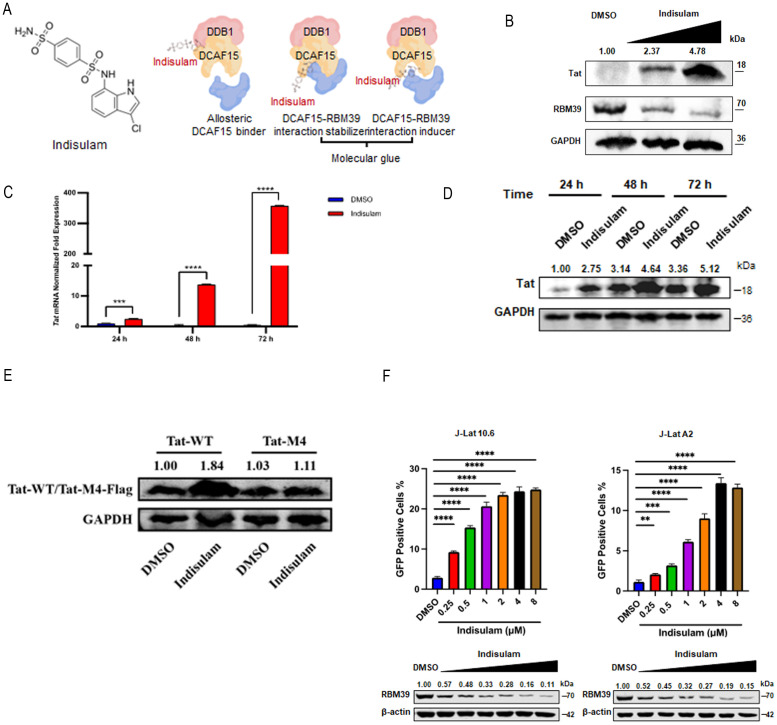
RBM39 participates in the establishment and maintenance of HIV-1 latency. **(A)** Chemical structure of *indisulam* (left panel). *Indisulam* functions by enhancing the interaction between the RBM39 splicing factor and the CUL4-DCAF15 E3 ubiquitin ligase complex, thereby inducing polyubiquitination and proteasomal degradation of RBM39 (right panel). **(B)** HEK293T cells were transfected with Tat-Flag plasmids. At 24 hours post-transfection, cells were treated with increasing concentrations of indisulam, and the expression levels of Tat-Flag and endogenous RBM39 were analyzed by western blotting. **(C, D)** HEK293T cells were transfected with Tat-Flag plasmids. At 24 hours post-transfection, cells were treated with *indisulam* (1 µM). Relative Tat mRNA levels were quantified using RT-qPCR (C), and Tat-Flag expression was assessed by western blotting (D). Data are presented as means ± SEM from triplicate measurements. *P*-values were calculated using an unpaired Student *t* test. ***P < 0.001; ****P < 0.0001. **(E)** HEK293T cells were transfected with WT Tat-Flag or M4 Tat-Flag plasmids. At 24 hours post-transfection, cells were treated with *indisulam* (1 µM) for 48 hours, after which the expression levels of WT Tat-Flag and M4 Tat-Flag were analyzed by western blotting. **(F)** J-Lat 10.6 and J-Lat A2 cells were treated with increasing concentrations of *indisulam*. Twenty-four hours post-treatment, the percentage of GFP-positive cells was analyzed by flow cytometry (upper panel), and endogenous RBM39 expression was quantified by western blotting (lower panel). Data are presented as means ± SEM from triplicate measurements. *P*-values were calculated using an unpaired Student *t* test. **P < 0.01; ***P < 0.001; ****P < 0.0001. The underlying data for this figure can be found in the ‘Raw Data’ file in https://doi.org/10.5281/zenodo.17470926.

To translate our findings into a potential therapeutic strategy, we leveraged *indisulam*, a clinical-stage molecular glue that induces the degradation of RBM39 by recruiting it to the E3 ligase DCAF15 [35] (Fig 5A). Due to its ability to degrade RBM39, *indisulam* has emerged as a promising candidate for cancer therapy. For instance, in liver cancer, elevated arginine levels regulate the transcription of metabolism-related genes by binding to RBM39, thereby modulating metabolic reprogramming and promoting tumor progression. Additionally, *indisulam* has been shown to inhibit tumor growth in a dose-dependent manner [36]. While *indisulam*’s anticancer activity is well-documented, its potential to disrupt HIV-1 latency remained unexplored.

We first confirmed the degradation-inducing effect of *indisulam* on RBM39 in HEK293T cells. The results showed that *indisulam* inhibited the expression of RBM39 protein in a dose-dependent manner while concomitantly promoting the expression of Tat (Fig 5B). To further characterize this effect, we treated cells with *indisulam* and assessed Tat expression at 24-, 48-, and 72-hours post-treatment using western blot and RT-qPCR. Time-course experiments revealed that *indisulam* increased Tat protein and mRNA levels as early as 24 hours post-treatment (Fig 5C and 5D). Consistent with our RBM39 knockdown experiments, *indisulam* increased the expression of wt Tat but failed to affect the expression of the m⁶A-site-deficient Tat mutant (Fig 5E).

Next, we evaluated whether *indisulam* treatment was as effective as RBM39 depletion in reactivating latent pseudotyped HIV-1. J-Lat 10.6 and J-Lat A2 cells were treated with *indisulam* at concentrations ranging from 0.25 to 8 μM to establish a dose–response relationship. Flow cytometry analysis revealed that *indisulam* reactivated latent pseudotyped HIV-1 in a dose-dependent manner, while western blot results confirmed the concomitant inhibition of RBM39 in both cell lines (Figs 5F and S2). Furthermore, *indisulam* treatment failed to significantly induce HIV-1 expression in RBM39-KO J-Lat 10.6 cells (S2 Fig), indicating that its activation potential is substantially dependent on RBM39.

To explore the potential of combination therapies for reactivating latent HIV-1, J-Lat 10.6 and J-Lat A2 cells were treated with Bryostatin-1 (PKC agonist), JQ-1 (BET inhibitor), or SAHA (HDAC inhibitor) alone or in combination with *indisulam*. Flow cytometry analysis demonstrated that combining *indisulam* with Bryostatin-1, JQ-1, or SAHA significantly increased the proportion of cells with reactivated latent HIV-1 compared to single-agent treatments (S3 Fig). These results position RBM39 degradation as a promising strategy to enhance the breadth and efficacy of “shock and kill” approaches.

### *Indisulam* reactivate diverse HIV-1 proviruses in primary cells

**Fig 6:**
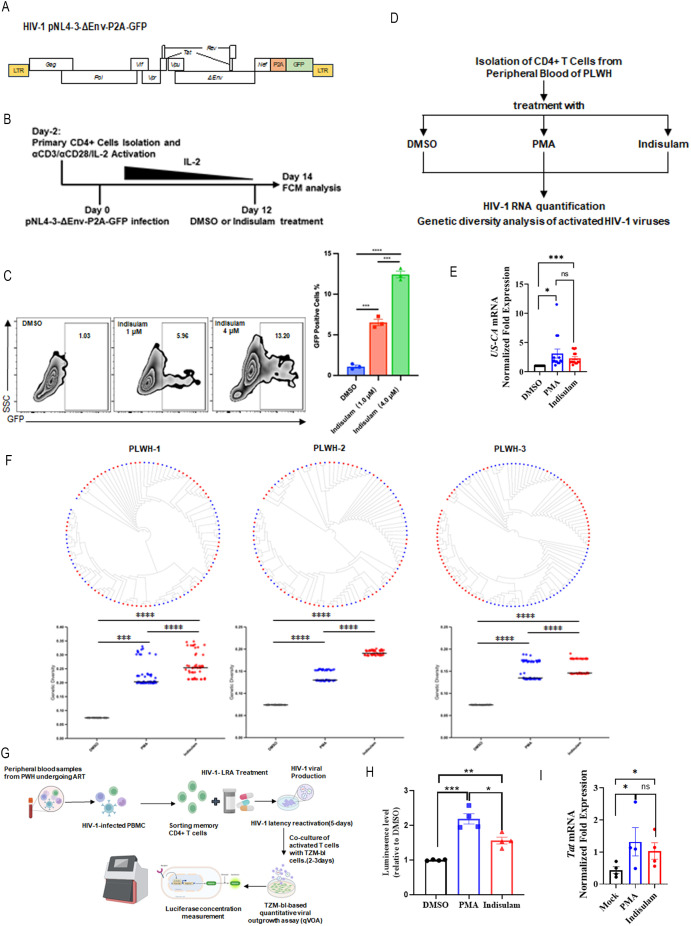
Indisulam reactivates diverse HIV-1 proviruses. **(A)** Schematic representation of the pseudotyped HIV-1 pNL4-3-ΔEnv-P2A-GFP backbone. **(B)** Experimental procedure for indisulam-mediated HIV-1 reactivation in primary CD4+ T cells. **(C)** The percentage of GFP-positive cells was analyzed by flow cytometry (left panel). Corresponding statistical results are presented in the right panel. Data are presented as means ± SD from triplicate measurements. *P*-values were calculated using an unpaired Student *t* test. ***P < 0.001; ****P < 0.0001. **(D)** Procedure for HIV-1 reactivation in primary CD4+ T cells isolated from people living with HIV (PLWH). **(E)** CD4+ T lymphocytes were isolated from PBMCs collected from 14 individuals living with HIV-1 (PLWH) who were receiving long-term antiretroviral therapy (ART). Cells were subsequently treated with DMSO, PMA, or indisulam. After 48 hours, cell lysates were prepared for RNA extraction, and viral RNA levels were quantified using real-time reverse transcription-PCR. Data are presented as mean ± SEM (*n* = 14 per group). Statistical analysis was performed using an unpaired Student *t* test. *P < 0.05; ***P < 0.001; ns, not significant. **(F)** Assessment of HIV-1 genetic diversity following activation with PMA or indisulam. HIV-1 strains were sampled from 3 virally activated subjects treated with PMA (blue) or indisulam (red). A total of 50 clones were sequenced per sample. Bootstrap consensus phylogenetic trees were constructed based on HIV-1 sequences obtained from PLWH. Strains were sampled from subjects treated with DMSO (black), PMA (blue), or indisulam (red). Each point represents the genetic distance between a single clone and the overall population; the horizontal bar indicates the mean genetic distance. Data are presented as mean ± SEM/SD.*P*-values were calculated using an unpaired Student *t* test. *P < 0.05; ***P < 0.001; ****P < 0.0001. **(G)** Schematic overview of the TZM-bl-based viral outgrowth assay (qVOA) using resting memory CD4+ T cells isolated from HIV–positive individuals undergoing ART. **(H)** Quantification of HIV-1 reactivation by luciferase assay using samples from four donors. Cells were treated with DMSO (black), PMA (blue), or indisulam (red) at a concentration of 5 μM. Data are shown as mean ± SEM (*n* = 4 per group). Statistical significance was determined using an unpaired Student *t* test. *P < 0.05; **P < 0.01; ***P < 0.001. **(I)** Cells from (H) were lysed for RNA extraction, and Tat RNA levels were quantified by qPCR. Data are presented as mean ± SEM (*n* = 4 per group). Statistical significance was determined using One-tailed unpaired Student *t* test. *P < 0.05; ns, not significant. The underlying data for this figure can be found in the ‘Raw Data’ file in h*tt*ps://doi.org/10.5281/zenodo.17470926.

To validate the role of RBM39 in maintaining HIV-1 latency in primary cells, we established a latency model using primary CD4⁺ T cells infected with pseudotyped HIV-1 pNL4-3-ΔEnv-P2A-GFP, which harbors a near-full-length HIV-1 genome (Fig 6A). Cells were activated with αCD3/αCD28 antibodies and IL-2, infected two days post-activation, and cultured under progressively reduced IL-2 concentrations to mimic latency. Twelve days post-infection, cells were treated with DMSO (control) or *indisulam* (Fig 6B). The concentrations of 1 and 4 µM *indisulam* were selected for treatment in the latency model based on the concentration of *indisulam* that exerts an activating effect in J-Lat 10.6 (data shown in Fig 5F). Flow cytometry revealed that *indisulam* significantly increased the proportion of GFP⁺ cells, confirming RBM39’s role in maintaining viral quiescence (Fig 6C). To assess clinical relevance, we isolated CD4⁺ T cells from 14 antiretroviral therapy (ART)-suppressed individuals living with HIV-1 (PLWH) and treated them with DMSO, PMA (positive control), or *indisulam* (Fig 6D). *Indisulam* induced HIV-1 RNA levels comparable to PMA (Fig 6E), demonstrating its potency in reactivating patient-derived latent reservoirs. To evaluate the breadth of proviral reactivation, we performed phylogenetic analysis of HIV-1 *envelope* (V1–V3) sequences from CD4⁺ T cells of PLWH treated with PMA or *indisulam*. Both treatments reactivated genetically diverse quasispecies, as evidenced by distinct clustering in neighbor-joining trees. Genetic diversity analysis reveals that the genetic variability within HIV-1 quasispecies activated by *indisulam* is comparable to, or potentially higher than, that observed following PMA stimulation (Fig 6F).

We also performed a TZM-bl cell-based quantitative viral outgrowth assay on memory CD4⁺ T cells isolated from patients on long-term cART. Our results demonstrate that indisulam treatment significantly enhances the activation of the replication-competent HIV-1 reservoir (Fig 6G and 6H). We further assessed Tat gene expression in these memory CD4⁺ T cells before and after activation, revealing that indisulam treatment markedly increases Tat RNA levels (Fig 6I). These findings indicate that residual Tat transcription exists in clinical latent reservoirs despite suppressive ART and that indisulam reactivates HIV-1 by elevating Tat RNA expression beyond the threshold required to initiate the positive feedback activation loop.

In conclusion, our results demonstrate that *indisulam* effectively reactivates a broad spectrum of genetically distinct HIV-1 proviruses. By degrading RBM39 and stabilizing Tat expression, *indisulam* provides a novel mechanism to disrupt viral latency, positioning it as a promising candidate for combinatorial “shock and kill” regimens.

## Discussion

**Fig 7:**
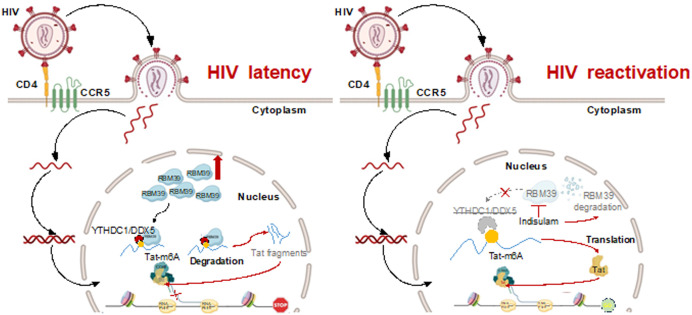
The schematic diagram. A schematic diagram showing two models of HIV-1 infection. Left panel (HIV-1 latency model): Under HIV-1 infection, upregulated RBM39 promotes m⁶A methylation of Tat RNA. This modification is recognized by YTHDC1, which subsequently recruits the RNA helicase DDX5 to degrade m⁶A-methylated Tat RNA. As a result, Tat RNA fails to undergo splicing and translation into functional Tat protein, thereby preventing activation of the HIV-1 LTR promoter and maintaining viral latency. Right panel (HIV-1 reactivation model): Treatment with *indisulam* induces degradation of RBM39. This abolishes the recognition of Tat RNA by YTHDC1 and its degradation by DDX5. Consequently, Tat RNA is efficiently translated into Tat protein, which activates the HIV-1 LTR promoter, leading to HIV-1 reactivation.

Our study uncovers an epitranscriptomic axis—RBM39-YTHDC1-DDX5—that enforces HIV-1 latency through m⁶A-dependent Tat RNA decay (Fig 7). By demonstrating that RBM39 recruits YTHDC1 and DDX5 to accelerate Tat RNA decay, we bridge a critical gap in understanding how post-transcriptional RNA modifications regulate viral persistence. These findings position RBM39 as a central checkpoint in HIV-1 latency and a promising therapeutic target for reservoir eradication.

The discovery that RBM39 enhances m⁶A modifications on Tat RNA expands the functional repertoire of RBPs in viral latency. While m⁶A has been implicated in HIV-1 replication and immune evasion [18,37], its role in maintaining viral quiescence remained unexplored. Our work reveals that RBM39 acts as a scaffold, bridging METTL3-mediated m⁶A deposition on Tat RNA with YTHDC1-dependent recognition and DDX5-driven decay. This tripartite mechanism parallels recent findings in cancer biology, where DDX5 collaborates with METTL3 to regulate m⁶A-dependent RNA splicing [32], but represents the first report of such a pathway in viral latency. Notably, the dependence on YTHDC1’s aromatic cage for m⁶A recognition underscores the specificity of this interaction, distinguishing it from broader m⁶A-mediated regulatory networks involving cytoplasmic readers like YTHDF1-3 [30].

While didehydro-cortistatin A (dCA), a candidate “block-and-lock” therapeutic strategy, potently inhibits Tat function by preventing its recruitment of P-TEFb to TAR and thereby suppressing HIV promoter transcription [38–40], our study identifies RBM39 as an m⁶A-dependent governor of Tat mRNA stability—a pre-translational checkpoint. The size of the HIV-1 reservoir is potentially underestimated because some replication-competent proviruses are hard to be activated from the resting state, and the expression level of HIV-1 proviruses exhibits stochastic fluctuations, which may be driven by the concentration of the Tat protein [41]. This RBM39-mediated decay pathway likely interacts with the stochastic Tat-transactivation network—stochastic fluctuations in Tat expression appear sufficient to drive phenotypic bifurcation and may contribute to proviral latency [42,43]. By reducing Tat transcript abundance, RBM39 may attenuate the positive feedback loops that are essential for viral reactivation. In parallel, HIV-1 utilizes post-transcriptional negative feedback mechanisms, such as the auto-depletion of unspliced mRNA precursors, to reinforce latency maintenance and suppress transcriptional noise [44]. We propose that RBM39-mediated degradation of Tat mRNA constitutes an additional mechanism of noise suppression, acting at the level of transcript stability, upstream of Tat protein synthesis. The precise mechanism by which T-cell activation signals counteract this decay machinery remains to be fully elucidated; however, dysregulation of m⁶A regulatory factors (e.g., METTL3, FTO) in quiescent cells may represent a plausible underlying mechanism [27,45]. Collectively, modulation of Tat mRNA stability through RBM39 represents a distinct pre-translational strategy for latency reversal, which complements existing approaches such as dCA-mediated inhibition of Tat protein function.

The pharmacological degradation of RBM39 by *indisulam*, a clinically explored anticancer agent [11,35,46], reactivated latent HIV-1 in primary CD4⁺ T cells from PLWH with efficacy comparable to PMA. This finding is particularly compelling given *indisulam*’s ability to synergize with Bryostatin-1, JQ-1, and SAHA, suggesting that combinatorial regimens could overcome the heterogeneity of latent reservoirs. Unlike traditional LRAs, which primarily target chromatin-modifying enzymes or signaling pathways, *indisulam*’s mechanism—disrupting a host-driven RNA decay pathway—represents a paradigm shift in latency reversal.

While our study identifies RBM39 as a critical regulator of Tat mRNA stability through m⁶A-dependent decay, several important questions regarding context-specific regulation remain to be addressed. Our preliminary experimental data indicate that RBM39 protein expression is significantly upregulated during CD4⁺ T cell activation. We propose that activation-induced RBM39 upregulation may function as a pivotal upstream regulatory signal, enhancing the assembly or functional activity of the decay complex in activated cells, thereby promoting rapid degradation of Tat mRNA. Conversely, reduced RBM39 expression in resting cells may impair Tat mRNA decay efficiency, thereby contributing to its stabilization and the maintenance of viral latency. This observation offers a plausible mechanistic basis for the differential regulation of the decay axis according to cellular activation status. However, three critical dimensions require further investigation: First, whether RBM39 inhibition perturbs other latency-associated pathways, such as NF-κB or AP-1 signaling, given its reported interactions with these transcriptional regulators [12,13]. Second, while we focused on Tat, m⁶A modifications are widespread across the HIV-1 genome [47]; profiling their dynamics during latency and reactivation could identify conserved epitranscriptomic “hotspots” exploitable for broad-spectrum LRAs. Third, our findings in primary cells highlight the need to validate this mechanism in in vivo models, such as humanized mice or non-human primates, to assess reservoir reduction and safety. Moreover, the upstream drivers of RBM39 upregulation, including TCR signaling, post-translational modifications, and activation-dependent modulation of m⁶A writers/erasers, represent essential mechanistic nodes for future elucidation.

The RBM39-YTHDC1-DDX5 axis offers a dual therapeutic opportunity: reactivating latent HIV-1 while simultaneously sensitizing infected cells to immune clearance. For example, *indisulam*-induced Tat expression could enhance viral antigen presentation, synergizing with checkpoint inhibitors or therapeutic vaccines. Furthermore, monitoring m⁶A levels on Tat RNA might serve as a biomarker for reservoir activity or LRA efficacy. Beyond HIV-1, this work raises intriguing questions about whether similar epitranscriptomic checkpoints govern latency in other viruses, such as HSV or HBV, which also encode RNA-binding regulatory proteins [48,49].

## Materials and methods

### Ethics statement

All human samples were anonymously coded in accordance with the local ethical guidelines (as stipulated by the Declaration of Helsinki). The Ethics Review Board of Sun Yat-Sen University and the Ethics Review Board of Guangzhou 8th People’s Hospital approved this study. Written informed consent was obtained from all patients voluntarily prior to their inclusion in this study. The experimental protocol has been approved by the Institutional Review Board of Guangzhou Eighth People’s Hospital (license number: 202033166).

### Participants

Peripheral blood for the isolation of primary CD4 cells was obtained from HIV-1-infected individuals. HIV-1-infected individuals had been on cART for at least 12 months and had maintained undetectable HIV-1 viremia (<50 HIV-1 RNA copies per ml of plasma). All the HIV-1-infected individuals were recruited from Guangzhou Eighth People’s Hospital. Buffy coats derived from the blood of healthy donors were used in in vitro experiments.

### Cell culture

HEK293T and Jurkat cells were obtained from ATCC. TZM-bl cells (NIH AIDS Reagent Program, Cat#8129), J-Lat 10.6 cells (NIH AIDS Reagent Program, Cat#9849) and J-Lat A2 cells (NIH AIDS Reagent Program, cat#9867) constructed by Dr. Eric Verdin (The Buck Institute for Research on Aging, Novato, CA) Laboratory, were obtained from Robert F. Siliciano (Department of Medicine, Johns Hopkins University School of Medicine, Baltimore, MD, USA). HEK293T, TZM-bl cells were cultured in DMEM supplemented with 1% penicillin-streptomycin (ThermoFisher) and 10% fetal bovine serum (FBS) (ThermoFisher). Jurkat, J-Lat 10.6, J-Lat A2 cells, and primary CD4^+^ T cells isolated from healthy donors or PLWH were cultured in RPMI-1640 supplemented with 1% penicillin-streptomycin and 10% FBS. All cells were cultured in a sterile incubator at 37 ℃ and 5% CO_2_.

### Plasmids, shRNAs, and sgRNAs

The cDNA coding sequence regions of RBM39, YTHDC1, METTL3, METTL14, and DDX5 tagged with N-terminal HA or Flag were amplified by reverse transcription-PCR (RT-PCR), with the mRNA of Jurkat cells as the template, and then were subcloned into pcDNA3.1 vector. The m⁶A binding site mutated YTHDCl (YTHDC1-mut, referred to as W377A and W428A), YTHDC1-W377A/W428A, YTHDC1-1-354 aa, and YTHDC1-493-727 aa were constructed from pcDNA3.1-YTHDC1-HA by overlapping PCR. RBM39-1-328 aa, RBM39-329-530 aa, RBM39-∆RRM1, RBM39-∆RRM2, RBM39-∆RRM1-∆RRM2, and RBM39-∆AD mutants were constructed from pcDNA3.1-RBM39-HA by overlapping PCR. YTHDC1-355-492 aa sequence regions were constructed from pcDNA3.1-YTHDC1-HA and then inserted into the pEGFP-N1 vector. pcDNA3.1-Tat-HA/Flag and pcDNA3.1-ubiquitin-HA were preserved by our laboratory. Tat m⁶A mutants were constructed from pcDNA3.1-Tat-Flag by overlapping PCR. shRNAs targeting luciferase (shNT) and shRNAs targeting sequences against RBM39 were used in combination. Target sequences were subcloned into the pLKO.1-RFP shRNA expression vector, which was derived from pLKO.1-puro. The puromycin resistance gene was replaced by the red fluorescent protein (RFP) tag. Two sgRNA pairs targeting RBM39 and YTHDC1 were employed (sequences provided in S1 Table), along with a non-targeting sgRNA pair (sgNC) serving as a control to differentiate gene-specific KO effects from potential off-target artifacts. Target sequences were annealed and cloned into LentiCRISPR V2 vector using BsmBI overhangs and sequence-verified. All constructs above were verified by DNA sequencing.

### RNA pull-down

RNA pull-down was performed on J-Lat 10.6 cells using High-Capacity Streptavidin Agarose Resin (Thermo Scientific Pierce), according to the manufacturer’s instructions. The Tat biotinylated RNA probes were designed and synthesized by Ruibiotech (sequences provided in S2 Table). The control RNA probes used in this study was obtained from Gong and colleagues [50]. Approximately 1 × 10^7^ J-Lat 10.6 cells and 50 µL streptavidin beads were used for each sample. The biotinylated RNA probes were incubated with agarose beads at 4 °C for 12 hours with rotation, after washing three times with binding buffer (0.1M phosphate, 0.15M sodium chloride). After washing the prepared cells with phosphate-buffered saline (PBS), 500 µL of cell lysis buffer was added. The mixture was supplemented with RNasin Ribonuclease Inhibitor and a Protease Inhibitor Cocktail to prevent degradation of nucleic acids and proteins, respectively. The lysate was then incubated on ice for 30 min and centrifuged at 14,000*g* for 5 min. The supernatants were collected and co-incubated with the RNA-bead complexes at 4 °C for 4 hours with rotation. Post-incubation, the agarose beads were pelleted by centrifugation, washed, and then resuspended with 80 µL elution buffer and 20 µL 5 × loading buffer and heated at 100 °C for 10 min. Following the heating step, the supernatant was carefully harvested and utilized as the sample source for subsequent western blot analysis. The samples were analyzed by 10% SDS-PAGE and visualized by Fast Silver Stain Kit (P0017s, Beyotime) according to the manufacturer’s instructions. After staining, the proteins were extracted from the gel bands and prepared for mass spectrometry analysis to identify and characterize the protein profiles.

### Gene Ontology (GO) enrichment analysis

GO enrichment analysis was performed on the 181 Tat-related proteins identified in J-Lat 10.6 cells using the clusterProfiler R package [51]. The analysis was conducted using the biological process category of GO terms. *P*-values were adjusted using the Benjamini–Hochberg method to control the false discovery rate. Only terms with an adjusted *p*-value <0.05 were considered significant. The results were visualized using the ggplot2 R package, where the size of dots represents the count of genes in each pathway and the color indicates the significance level.

### Protein–protein interaction network construction and functional enrichment analysis

Protein–protein interaction networks were constructed using the STRING database (version 12.0) with the 181 Tat-related proteins identified from proteomic analysis [52]. In this complex PPI network, hub genes were defined as highly interconnected nodes [53]. Network visualization and clustering analysis were performed using Cytoscape (version 3.10.3). K-means clustering was applied with *k* = 12 to identify functionally related protein modules within the network. Bottleneck centrality analysis was performed using the CytoHubba plugin in Cytoscape to rank proteins based on their network importance [54], and the top 20 ranked proteins were selected for detailed interaction analysis. KEGG pathway enrichment analysis was performed using the STRING database for each protein cluster. Results were visualized as horizontal bar charts showing the number of genes associated with each pathway category.

### HIV-1 latency model construction

The CD4^+^ T cell latency models were established using the pseudotyped HIV-1 clone HIV-1 NL4-3-ΔEnv-P2A-GFP. This clone was derived from the wt HIV infectious clone pNL4-3 by deleting a portion of the env gene and inserting an ORF containing P2A and GFP downstream of the nef gene. A total of 3 μg of VSV-G envelope plasmid and 9 μg of pseudotyped virus construct was co-transfected into HEK293T cells. Forty-eight hours post-transfection, the virus supernatants were concentrated with PEG6000 and used to infect CD4^+^ T cells. Primary CD4^+^ T cells were isolated from healthy donor peripheral blood mononuclear cells and activated with αCD3/αCD28 antibodies and IL-2. The activated cells were then infected with the concentrated pseudotyped virus. After infection, the cells were cultured in a medium with decreasing concentrations of IL-2. Finally, cell lysates were subjected to PCR amplification using HIV-1 gag SK38/SK39 primers (sequences listed in S3 Table), and HIV-1 reactivation was confirmed by flow cytometry analysis.

### Genetic diversity analysis of activated HIV-1 viruses

The genetic diversity of HIV-1 quasispecies under different treatments was evaluated through the sequencing of the env V1-V3 segment. HIV-1 RNAs from each group were reverse-transcribed by specific primer ES8B. The V1-V3 region was amplified using a two-step nested PCR approach with the following primer combinations: E00 forward primer and ES8B reverse primer for the first round, followed by E20 forward primer and E115 reverse primer for the second round (sequences provided in S4 Table) [55]. Following two rounds of nested PCR using Phanta Max Master Mix (Vazyme), the resulting amplicons underwent deoxyadenosine (dA) tailing at their 3′ ends using Ex Taq DNA polymerase (Takara). The dA-tailed PCR products were subsequently TA cloned into the pMD-18T vector. To reduce the risk of sampling bias, a method for amplifying single genomes was employed. For each sample, 50 distinct PCR products were cloned to ensure a diverse representation of the viral genome. The alignments of HIV-1 sequences were built by using ClustalW, and all ambiguous positions were removed for each sequence pair using the pairwise deletion method [56]. Subsequently, the average genetic distance between each clone and the relevant entire population in each sample was calculated using MEGA 12 with Maximum Composite Likelihood model. To further depict the globe landscape of HIV diversity, the phylogenetic bootstrap consensus trees were constructed using the neighbor-joining method with 1,800 bootstrap replications implemented in MEGA 12 [57]. The resulting phylogenetic tree was then visualized and aesthetically enhanced using the Interactive Tree of Life (iTOL) online tool [58].

### Construction of gene knockout stable cell lines

To generate RBM39 KO HEK293T cell line, YTHDC1 KO HEK293T cell line and RBM39 KO J-Lat 10.6 cell line, HEK293T cells were transfected with 10 µg LentiCRISPR V2 carrying genes for sgRNAs that targeted RBM39 or YTHDC1, 5 μg of VSV-G envelope plasmid and 10 μg of lentiviral packaging plasmid psPAX2 (per 10 cm dish)were co-transfected into HEK293T cells using polyethylenimine (PEI, PEI/DNA ratio 3:1; Polyplus, 101000017) and lentiviruses were harvested and concentrated with PEG6000 and then used to infect target cells, including HEK293T and J-Lat 10.6 cell lines. Forty-eight hours after infection, the infected cells were selected in a medium supplemented with 2 μg/mL puromycin for 3 days (Sigma-Aldrich, P8833). Cells were cultured at 37 °C in a humidified atmosphere with 5% CO_2_. The knockdown efficiency was confirmed through western blot for RBM39 or YTHDC1 expression.

### RNA isolation and quantitative real-time PCR

Total cellular RNA was extracted using a EZ-press RNA Purification Kit (EZBioscience) and reverse transcribed into cDNA with a HiScript II 1st Strand cDNA Synthesis Kit (R211-02, Vazyme). Real-time PCR was conducted using ChamQ Universal SYBR qPCR Master Mix (Q711-02, Vazyme) in a CFX96 real-time PCR detection instrument (Bio-Rad). The data were analyzed by a SYBR green-based system (Bio-Rad), semiquantified, and normalized to GAPDH/β-Actin or quantified with the help of a standard curve. The primers used are listed in S5 Table.

### RNA stability assay

To assess Tat RNA stability, cells were incubated with ActD to terminate transcription. Briefly, HEK293T cells were incubated with ActD (5 µg/ml) and collected. Total RNA was extracted, and the Tat RNA expression was determined by RT-qPCR.

### RNA immunoprecipitation (RIP)

The RIP assay was conducted according to the manufacturer’s instructions (BersinBio, China). Briefly, the cells were lysed to remove DNA, and the remaining lysate was divided into input, IgG, and IP groups. A total of 5 μg of IgG and primary antibody was added to the IgG and IP groups, respectively, and incubated overnight at 4 °C. Sixteen hours after incubation, 20 µL protein A/G magnetic beads were added to the IgG and IP groups, respectively, and incubated at 4 °C for 1 h. The magnetic beads were collected using a magnetic holder and washed twice. Finally, the RNA-protein conjugated to the magnetic beads was eluted and TRIzol reagent (ThermoFisher) was used for RNA extraction and purification. RT-qPCR was used to detect the expression levels of the target genes were performed as previously described.

### RNA m⁶A quantification

The m⁶A modification level of total RNA was conducted via EpiQuik m⁶A RNA Methylation Quantification Kit (P-9005) according to the manufacturer’s instruction. Briefly, 200 ng RNA accompanied with m⁶A standard were coated on assay wells, followed by capture antibody solution and detection antibody solution. The m⁶A levels were quantified colorimetrically by reading the absorbance of each well at a wavelength of 450 nm (OD450), and then calculations were performed based on the standard curve.

### Co-immunoprecipitation and western blot

HA-tagged and Flag-tagged plasmids were transfected into HEK293T cells. Forty-eight hours post-transfection cells were harvested and lysed in ice-cold NP-40 lysis buffer (10 mM Tris-HCl buffered at pH 7.5, 150 mM NaCl, 0.5% NP-40, 1% Triton X-100, 10% Glycerol, 2 mM EDTA, 1 mM NaF, 1 mM Na3VO4). Cell lysates were incubated with anti-Flag agarose beads (M8823, Sigma-Aldrich) or Protein A/G PLUS-Agarose (sc-2003 SANTA CRUZ) with the specified immunoprecipitating antibodies while rotating overnight. Then the beads were collected by centrifuging and removing the supernatant. The beads were washed five times again with ice-cold STN buffer. IP samples were eluted by boiling with 5 × protein SDS-PAGE loading buffer at 100 ℃ for 10 min. Western blots were conducted with indicated antibodies. GAPDH or β-actin was used as internal reference. The secondary antibodies were 680RD goat anti-mouse IgG antibody and 800CW goat anti-rabbit IgG antibody. Images were acquired with an Odyssey CLX imager and analyzed by Image Studio Lite, v.4.0.

### Mass spectrometry

To identify Tat interaction components in the presence or absence of RBM39, Tat-Flag was overexpressed with Flag-tagged pcDNA3.1-Flag (control) or RBM39-Flag in HEK293T cells. Forty-eight hours post-transfection, cells were harvested and IP with anti-Flag beads. Enriched proteins were washed and eluted by boiling with 5 × protein SDS-PAGE loading buffer. Proteins of each sample were separated by 4%–12% gradient SDS-PAGE gel. Eight slices were cut out for each sample. The in-gel digestion and LC–MS/MS analysis were the same as GFP-Flag control samples. The generated raw data was annotated and analyzed utilizing PEAKS Studio.

### Transfection of siRNA and plasmids

siRNAs targeting indicated human genes, and negative control siRNA (siNC) were purchased from RiboBio (Guangzhou, China). The primers sequences are listed in S6 Table. Three siRNAs were synthesized for each gene. The siRNAs targeting each gene were transfected as a mixture and have been validated by the company to ensure that at least one siRNA was able to knockdown target gene mRNA up to 70%. HEK293T cells were transfected with specific siRNAs targeting each gene using Lipofectamine RNAiMAX (ThermoFisher) according to the manufacturer’s instruction when the cell density reached approximately 60%. Each gene was set three biological replicates. At 48 hours post-transfection, cells were collected for western blot.

### Flow cytometry

Cells were harvested after infection or treatment, then washed twice with PBS and resuspended. Each group of the GFP-positive-infected cells were detected using BD LSR Fortessa flow cytometer and analyzed with FlowJo V10 software. The percentage of RFP-positive cells indicated the efficiency of shRNA lentiviral infection.

### Antibodies

The following specific antibodies were used for RIP, Co-IP, or immunoblotting: normal rabbit anti-IgG antibody (2729; CST), anti-RBM39 antibody (21339-1-AP, Proteintech), anti-HA antibody (51064-2-AP, Proteintech), anti-Flag antibody (20543-1-AP, Proteintech), anti-β-actin (66009-1-Ig, Proteintech), anti-GAPDH antibody (10494-1-AP, Proteintech), anti-N^6^-methyladenosine antibody (A19841, Abclonal), anti-YTHDC1 antibody (ab264375, Abcam), anti-METTL3 antibody (ab195352, Abcam), anti-ALKBH5 antibody (16837-1-AP, Proteintech), anti-GFP antibody (50430-2-AP, Proteintech), IRDye 680RD goat anti-mouse IgG antibody (926-68070; LI-COR Biosciences), and IRDye 800CW goat anti-rabbit IgG antibody (926-32211; LI-COR Biosciences).

### Data statistical analysis

All the statistical details of specific experiments, which included the statistical tests used, number of samples, mean values, standard errors of the mean (SEM) and *p*-values derived from indicated tests, had been described in the figure legends and showed in the figures. Statistical analyses were conducted utilizing GraphPad Prism. Triplicate data were presented as mean ± SEM. A value of *p* < 0.05 was considered to be statistically significant and represented as asterisk (*). Value of *p* < 0.01 was considered to be more statistically significant and represented as double asterisks (**). Value of *p* < 0.001 was considered to be the most statistically significant and represented as triple asterisks (***). Value of *p* < 0.0001 was considered to be more statistically significant and represented as double asterisks (****). For comparison between two treatments, a Student *t* test was used. For the comparison of highly he*t*erogeneous data including HIV-1 reactivation of clinical samples and genetic diversity index experiment, a Mann–Whitney *U* test was used.

## Supporting information

S1 Fig YTHDC1 facilitates Tat degradation. HEK293T cells were co-transfected with Tat-Flag and plasmids expressing YTHDF1-HA, YTHDF2-HA, YTHDF3-HA, YTHDC1-HA, METTL3-HA, FTO-HA, or ALKBH5-HA. At 48 hours post-transfection, cells were lysed and subjected to western blot analysis using the indicated antibodies. The underlying data for this figure can be found in the ‘Raw Data’ file in https://doi.org/10.5281/zenodo.17470926.
(TIF)

S2 Fig Effects of RBM39 knockdown and indisulam treatment on J-Lat cells. J-Lat A2, J-Lat 10.6, and RBM39 KO J-Lat 10.6 cells were treated with increasing concentrations of indisulam. Twenty-four hours post-treatment, the percentage of GFP-positive cells was analyzed by flow cytometry. The underlying data for this figure can be found in the ‘Raw Data’ file in https://doi.org/10.5281/zenodo.17470926.
(TIF)

S3 Fig Indisulam synergizes with other latency-reversing agents in reactivating HIV-1 latency. **(A)** J-Lat 10.6 cells were treated with Bryostatin-1 (10 μM), JQ-1 (1 μM), or SAHA (500 nM) alone, in combination with indisulam, or with DMSO as a control. Twenty-four hours post-treatment, the percentage of GFP-positive cells was quantified by flow cytometry. Data are presented as means ± SEM from triplicate experiments. *P*-values were calculated using an unpaired Student *t* test. **P < 0.01; ***P < 0.001; ****P < 0.0001. **(B)** J-Lat A2 cells were treated with Bryostatin-1 (10 μM), JQ-1 (1 μM), or SAHA (500 nM) alone, in combination with indisulam, or with DMSO as a control. Twenty-four hours post-treatment, the percentage of GFP-positive cells was quantified by flow cytometry. Data are presented as means ± SEM from triplicate experiments. *P*-values were calculated using an unpaired Student *t* test. **P < 0.01; ***P < 0.001; ****P < 0.0001. The underlying data for this figure can be found in the ‘Raw Data’ file in https://doi.org/10.5281/zenodo.17470926.
(TIF)

S1 Table Primers for shRNA/sgRNA plasmid construction. (PDF)

S2 Table Biotinylated RNA probes used for RNA pull down. (PDF)

S3 Table Primers for HIV US-CA RNA identification. (PDF)

S4 Table Primers for nested PCR (amplifying the V1–V3 region of HIV-1 *envelope*). (PDF)

S5 Table Primers for RT-qPCR. (PDF)

S6 Table siRNAs used for gene silencing (target sequence). (PDF)

## Abbreviations

ActD: actinomycin D
AIDS: Acquired Immune Deficiency Syndrome
ART: antiretroviral therapy
CTD: C-terminal domain
dCA: didehydro-cortistatin A
FBS: fetal bovine serum
GO: Gene Ontology
HIV-1: Human Immunodeficiency Virus 1
iTOL: Interactive Tree of Life
KO: knockout
LC–MS/MS: liquid chromatography–mass spectrometry
LRAs: latency-reversing agents
LTR: long terminal repeat
NTD: N-terminal domain
PBS: phosphate-buffered saline
PKM: pyruvate kinase M
PLWH: people living with HIV-1
P-TEFb: positive transcription elongation factor b
RBM39: RNA-binding Motif Protein 39
RBPs: RNA-binding proteins
RFP: red fluorescent protein
RIP: RNA immunoprecipitation
RT-PCR: reverse transcription-polymerase chain reaction
RT-qPCR: reverse transcription quantitative polymerase chain reaction
SEM: standard errors of the mean
TAR: *trans*-activation response element
wt: wild-type
