# Supplementary material for: The RNA-binding protein RBM39 scaffolds an m⁶A-dependent RNA decay complex that destabilizes Tat transcripts and restricts HIV-1 reactivation
Source: PLoS Biol. 2025 Nov 11;23(11):e3003486. doi: 10.1371/journal.pbio.3003486 (PMC12617877; doi:10.1371/journal.pbio.3003486)
Supplement: S1 Table — (PDF) [file pbio.3003486.s004.pdf]

S1_ Table. Primers for shRNA/sgRNA plasmid construction

shNT	Forward primer (5'-3')<br>CCGGACCGCCTGAAGTCTCTGATTAACTCGAGTTAATCAGAGACTT<br>CAGGCGGTTTTTTG<br>Reverse primer (5'-3')<br>AATTCAAAAAACCGCCTGAAGTCTCTGATTAACTCGAGTTAATCAGA<br>GACTTCAGGCGGT
shRBM39	Forward primer (5'-3')<br>CCGGGCCGTGAAAGAAAGCGAAGTACTCGAGTACTTCGCTTTCTTT<br>CACGGCTTTTTG<br>Reverse primer (5'-3')<br>AATTCAAAAAGCCGTGAAAGAAAGCGAAGTACTCGAGTACTTCGCT<br>TTCTTTCACGGC
sgNC	Forward primer (5'-3'): CGCGATAGCGCGAATATATT<br>Reverse primer (5'-3'): AATATATTCGCGCTATCGCG
sgRBM39	Forward primer (5'-3'): AGCGAAGTAGAAGCAAAGAG<br>Reverse primer (5'-3'): CTCTTTGCTTCTACTTCGCT
sgYTHDC1	Forward primer (5'-3'): ATTCTTATAAGGTTCTCTGG<br>Reverse primer (5'-3'): CCAGAGAACCTTATAAGAAT
