# Supplementary material for: The RNA-binding protein RBM39 scaffolds an m⁶A-dependent RNA decay complex that destabilizes Tat transcripts and restricts HIV-1 reactivation
Source: PLoS Biol. 2025 Nov 11;23(11):e3003486. doi: 10.1371/journal.pbio.3003486 (PMC12617877; doi:10.1371/journal.pbio.3003486)
Supplement: S3 Table — (PDF) [file pbio.3003486.s006.pdf]

S3_ Table. Primers for HIV US-CA RNA identification

SK38	5'-3': ATAATCCACCTATCCCAGTAGGAGAAA
SK39	5'-3': TTTGGTCCTTGTCTTATGTCCAGAATGC
