# Supplementary material for: The RNA-binding protein RBM39 scaffolds an m⁶A-dependent RNA decay complex that destabilizes Tat transcripts and restricts HIV-1 reactivation
Source: PLoS Biol. 2025 Nov 11;23(11):e3003486. doi: 10.1371/journal.pbio.3003486 (PMC12617877; doi:10.1371/journal.pbio.3003486)
Supplement: S4 Table — (PDF) [file pbio.3003486.s007.pdf]

S4_ Table. Primers for Nested PCR (Amplifying the V1-V3 region of HIV-1 *envelope*)

1st-F-E00	5'-3': ATAATCCACCTATCCCAGTAGGAGAAA
1st-R-ES8B	5'-3': TTTGGTCCTTGTCTTATGTCCAGAATGC
2nd-F-E20	5'-3': GGGCCACACATGCCTGTGTACCCACAG
2nd-R-E115	5'-3': AGAAAAATTCCCCTCCACAATTAA
