# Supplementary material for: The RNA-binding protein RBM39 scaffolds an m⁶A-dependent RNA decay complex that destabilizes Tat transcripts and restricts HIV-1 reactivation
Source: PLoS Biol. 2025 Nov 11;23(11):e3003486. doi: 10.1371/journal.pbio.3003486 (PMC12617877; doi:10.1371/journal.pbio.3003486)
Supplement: S5 Table — (PDF) [file pbio.3003486.s008.pdf]

S5_ Table. Primers for RT-qPCR

Genes	Forward primer (5'-3')	Reverse primer (5'-3')
GAPDH	GATTCCACCCATGGCAAATTC	CTGGAAGATGGTGATGGGATT
β-actin	GCATGGAGTCCTGTGGCA	CAGGAGGAGCAATGATCTTGA
RBM39	GCAAGGACAGTCTTCTGTATGC	CGACGAACTCCACATAAGCAA
Tat	ATGGAGCCAGTAGATCCTAGACTAGAG	CGTCGCTGTCTCCGCTTCTTCCT
YTHDC1	AACTGGTTTCTAAGCCACTGAGC	GGAGGCACTACTTGATAGACGA
