# Supplementary material for: The RNA-binding protein RBM39 scaffolds an m⁶A-dependent RNA decay complex that destabilizes Tat transcripts and restricts HIV-1 reactivation
Source: PLoS Biol. 2025 Nov 11;23(11):e3003486. doi: 10.1371/journal.pbio.3003486 (PMC12617877; doi:10.1371/journal.pbio.3003486)
Supplement: S6 Table — (PDF) [file pbio.3003486.s009.pdf]

S6_ Table. siRNAs used for gene silencing (Target sequence)

siRBM39_1	GAAGCGAAGTAGAGACAGA
siRBM39_2	GACAGAAATTAAGACGTT
siRBM39_3	GGAAAGGACTGGAATTGAT
